# The protein kinase TOUSLED facilitates RNAi in *Arabidopsis*

**DOI:** 10.1093/nar/gku422

**Published:** 2014-06-11

**Authors:** Mohammad Nazim Uddin, Patrice Dunoyer, Gregory Schott, Salina Akhter, Chunlin Shi, William J. Lucas, Olivier Voinnet, Jae-Yean Kim

**Affiliations:** 1 Division of Applied Life Science (BK21+/WCU program), PMBBRC, Graduate School of Gyeongsang National University, Jinju 660-701, Korea; 2 Institut de Biologie Moléculaire des Plantes du CNRS, UPR2357, Université de Strasbourg, Strasbourg Cedex, France; 3 Department of Plant Biology, College of Biological Sciences, University of California, Davis, CA 95616, U.S.A.; 4 Department of Biology, Swiss Federal Institute of Technology (ETH), 8092 Zurich, Switzerland

## Abstract

RNA silencing is an evolutionarily conserved mechanism triggered by double-stranded RNA that is processed into 21- to 24-nt small interfering (si)RNA or micro (mi)RNA by RNaseIII-like enzymes called Dicers. Gene regulations by RNA silencing have fundamental implications in a large number of biological processes that include antiviral defense, maintenance of genome integrity and the orchestration of cell fates. Although most generic or core components of the various plant small RNA pathways have been likely identified over the past 15 years, factors involved in RNAi regulation through post-translational modifications are just starting to emerge, mostly through forward genetic studies. A genetic screen designed to identify factors required for RNAi in *Arabidopsis* identified the serine/threonine protein kinase, TOUSLED (TSL). Mutations in *TSL* affect exogenous and virus-derived siRNA activity in a manner dependent upon its kinase activity. By contrast, despite their pleiotropic developmental phenotype, *tsl* mutants show no defect in biogenesis or activity of miRNA or endogenous *trans*-acting siRNA. These data suggest a possible role for TSL phosphorylation in the specific regulation of exogenous and antiviral RNA silencing in *Arabidopsis* and identify TSL as an intrinsic regulator of RNA interference.

## INTRODUCTION

Ribonucleic acid (RNA) silencing is triggered by double-stranded RNA (dsRNA), processed into 21- to 24-nt small interfering (si)RNA or micro (mi)RNA by RNaseIII-like enzymes called Dicers, or Dicer-like (DCL) in plants (1–3). Small RNAs guide ARGONAUTE (AGO)-containing RNA-induced silencing complexes (RISCs) to suppress target gene expression at the level of transcription, RNA stability or translation. In *Arabidopsis*, 21-nt siRNA and miRNA mainly incorporate into AGO1 to promote cleavage or translational inhibition of target transcripts (4,5), whereas 24-nt siRNAs guide heterochromatin formation by recruiting AGO4, AGO6 or AGO9 (6). In higher plants, the effect of RNAi can also extend beyond the sites of its initiation, owing to the movement of signal molecules with defensive and developmental roles (7,8). The mobile signal is at least composed of siRNAs because these are necessary and sufficient to convey the sequence specificity of cell-to-cell and long distance silencing spread (9–11). The spread of virus-derived siRNA (vsiRNA) most likely immunizes surrounding cells that are yet to be infected (12), whereas movement of endogenous *trans*-acting (ta)siRNA generates a gradient of target gene expression participating in organ polarization (13,14).

Several genetic screens have been conducted to uncover genes required for RNAi or its non-cell-autonomous aspects in plants. The identified factors include the DCLs that hierarchically generate the various small RNAs, the AGOs into which small RNAs are loaded, as well as several cofactors required for efficient and/or accurate processing, protection or activity of small RNAs as part of the AGO or DCL ribonucleoprotein complexes (15). Several mutations have also been shown to compromise silencing cell-to-cell and/or long-distance spread through still highly elusive mechanisms. These include the largest subunit of the plant-specific heterochromatic NUCLEAR RNA POLYMERASE-IVa (NRPD1a), the RNA-DEPENDENT RNA POLYMERASE 2 (RDR2) or the SNF2 domain-containing protein, CLASSY1 (CLSY1) (16–18).

To identify new factors required for intracellular and/or non-cell-autonomous RNAi, we designed a sensitive transgenic system whereby siRNA production is driven by the phloem-restricted and moderately expressed, *AtSUC2* promoter. This system not only allows the identification of factors required for non-cell-autonomous RNAi but also of genes whose requirements in cell-autonomous RNAi may be bypassed when strong and constitutively expressed promoters, such as the *Cauliflower mosaic virus* 35S promoter, are used to express dsRNA inducers (16,19). Using this system in a forward genetic screen, we identified and characterized the serine/threonine protein kinase TOUSLED (TSL) (20). We show that TSL is required for RNAi in *Arabidopsis*, in a manner dependent upon its kinase activity. We further present evidence that TSL specifically affects exogenous and viral-derived siRNA activity without overt effect upon miRNA or endogenous tasiRNA biogenesis or activity.

## MATERIALS AND METHODS

### Plant materials

Mutant lines *dcl1-16* (Salk_013118), *dcl2-1* (Salk_064627), *nrpd1a-4* (Salk_083051), *nrpd1b-11* (Salk_029919), *tsl-8* (Salk_152957) and *tki1-1* (Salk_064187) were obtained from the Arabidopsis Biological Resource Center (ABRC). Mutant lines *dcl4-2, dcl3-1, rdr2-1, rdr6-15*, *ago1-27*, *asf1ab* and transgenic *SUC:SUL* (*SS*) line have been described previously (17,21). Genotyping of the Transfer DNA (T-DNA) insertion lines was performed by polymerase chain reaction (PCR), using allele-specific primers. PCR primers are listed in Supplementary Table S1. Seeds were surface-sterilized with 20% (v/v) commercial bleach and 0.1% (v/v) Triton X-100 for 5 min, washed three times with double distilled water and stored at 4°C for 3 days for stratification. Plants were grown under long-day conditions at 22°C with a 16 h light/8 h dark cycle, either in soil or on Murashige and Skoog (MS) agar plates.

### DNA constructs and plant transformation

The T-DNA expression cassette for firefly luciferase (*p35S:LUC*-3′ Nos) was cut with *Eco*RV, and inserted into the PmeI site of the binary vector pC1300 containing the hygromycin resistance gene. We constructed double-stranded luciferase (*dsLU*) by including the first 400 bp sequence from the start codon; a PCR amplified fragment was inserted into the XhoI/KpnI and XbaI/ClaI sites of pHannibal, respectively. The *dsLU* region containing the pyruvate dehydrogenase kinase (PDK) intron from pHannibal was transferred into the binary vector pC2300, containing the kanamycin resistance gene. The phloem-specific promoter, *pSUC2*, was amplified from *Arabidopsis* genomic DNA and inserted in front of the *dsLU*. For the *pSUC2:dsLU-p35S:LUC*, the *p35S:LUC* was inserted into the PmeI site of *pSUC2:dsLU*. Another construct (*pSUC2:GUS-dsLU-p35S:LUC*) was made with a β-glucuronidase (*GUS*) reporter fused downstream of the *AtSUC2* promoter.

For generating the *TSL^K438E^* mutation, the complementary DNA (cDNA) sequence for the catalytic domain with the codon for Lys-438 changed to a codon for Glu was generated by a PCR-based method (22). *TSL* and *TSL^K438E^* were cloned into the gateway entry vector (pENTR/D-TOPO) (Invitrogen) and inserts were confirmed by sequencing. The entry clones were subsequently transformed into gateway binary vector pEG101 to produce *p35S*:*TSL-YFP* and *p35S*:*TSL^K438E^-YFP*, respectively. As *tsl* mutants have a defective floral developmental phenotype, we used heterozygote *tsl* plants for all transformations. These constructs were transformed into *Agrobacterium tumefaciens* GV3101 and introduced by the floral dip method (23).

### Luminescence imaging

Both 7-day-old seedlings and plants at the rosette stage were sprayed with 1 mM luciferin (in 0.01% (v/v) Triton X-100) and kept in darkness for 4 min to allow full penetration of luciferin into the tissues. Luminescence images were acquired over a 100-s period using a pre-chilled charge-coupled device camera (−60°C) (ANDOR iXon Technology) (24).

### Ethyl methanesulfonate (EMS) mutagenesis

Surface-sterilized seeds (∼4000) from the homozygous transgenic plants carrying *pSUC2:dsLU-p35S:LUC* construct (*PL*) were exposed to 30 mM EMS for 12 h. Seeds were then rinsed five times with double distilled water, sowed uniformly on soil and cold-treated at 4°C for 3 days. M2 seeds were harvested as pools, with each pool containing 50 plants. Seeds (∼5000) from a single pool were surface-sterilized and sowed at a density of ∼500 seeds per plate for luminescence image screening.

### Genetic mapping

F2 mapping populations were obtained by crossing *impaired luciferase silencing 1* (*ils1*) plants (in the ecotype Col-0) to *Landsberg erecta*. Genomic DNA was isolated from F2 seedlings pre-screened for mutant phenotype and kanamycin resistance. An initial mapping population of 60 F2 plants, a series of simple sequence length polymorphism (SSLP) and cleaved amplified polymorphic sequence (CAPS) markers were used to determine the linkage of the *ils1* mutation within the short interval between the markers nga106 and nga139, on chromosome 5. Using the Monsanto *Arabidopsis* Polymorphism Database, we generated additional SSLP and CAPS markers between 6897 kb and 7369 kb. With an increased mapping population of 720 F2 plants, the *ils1* mutation was positioned to a 32-kb region (BAC f22d1), which contained several predicted genes. Genomic DNA sequencing of the candidate genes in the *ils1* mutant identified the mutation in *TSL*.

### Virus induced gene silencing (VIGS)

VIGS was carried out as previously described (25,26) with a *Tobacco rattle virus* (*TRV*) carrying a 500-bp insert corresponding to the *A. thaliana PHYTOENE DESATURASE* (*PDS*) sequence (*TRV-PDS*). The infectious clone of *TRV-PDS*, amplification of viral transcripts and inoculation of *Arabidopsis* plants were as previously described (26). Infected/photobleached systemic leaves were collected at 14 days post inoculation for RNA extraction.

### RT-PCR and qRT-PCR

Total cDNA was synthesized using the SuperScript III first-strand synthesis system for RT-PCR (Invitrogen), according to the manufacturer's instructions. For RT-PCR, PCR was performed on diluted cDNA using *Taq* polymerase (Solgent, South Korea). For qRT-PCR, we quantified the cDNA using the QuantiMix SYBR Kit (Philekorea Technology, South Korea) and gene-specific primers using the Eco™ Real-Time PCR system (Illumina), according to the manufacturer's protocol. Cycling conditions were as follows: 95°C for 10 min, followed by 40 cycles of 95°C for 10 s, 55°C for 40 s and 72°C for 15 s. For each cDNA synthesis, quantification was performed in triplicate. The *ACTIN2* was used as a reference as it is stably expressed across a wide range of conditions.

### GUS staining

GUS staining was performed on the T2 generations of transgenic lines, as previously described (27).

### Northern analysis

Total RNA was extracted from *Arabidopsis* rosette leaves with Trizol reagent (Sigma), according to the manufacturer's instructions. RNA gel blot analysis of high and low molecular weight RNA was performed with 10 and 30 μg of total RNA, respectively, and was as described previously (28). Ethidium bromide staining of total RNA, before transfer and U6 were used to confirm equal loading. RNA hybridizations were performed using the ULTRAHyb Oligo solution, according to the manufacturer's instructions (Ambion). Radiolabeled probes for detection of the *LUC*, *SUL* or *PDS* siRNAs were made by random priming reactions in the presence of [α-^32^P]-dCTP (Amersham). The template used was a 400 bp PCR product amplified from *LUC* cDNA. The 400 bp (for *SUL*) and 500 bp (for *PDS*) PCR products were amplified from *Arabidopsis* cDNA. DNA oligonucleotides complementary to miRNAs, tasiRNAs or hc-acting siRNAs were end-labeled with [γ-^32^P]-ATP using T4 polynucleotide kinase (New England Biolabs). All hybridization signals were detected by phosphor-imaging (Cyclone Plus Storage Phosphor System; PerkinElmer).

### Western analysis

Total proteins were isolated from either seedlings, rosette leaves of 1-month-old plants or flowers. Protein aliquots (20 μg) were resolved on an 8% SDS-PAGE gel, followed by electroblotting onto Immobilon-P membranes (Millipore). Membranes were blocked in Tris-buffered saline (TBS) buffer (25 mM Tris·HCl, pH 8.0, 125 mM NaCl) containing 5% skimmed milk for 1 h. Membranes were incubated with the respective antibodies diluted in TBS buffer (1:1000) containing 5% skimmed milk for 1 h. Blots were washed in TBS buffer containing 0.1% (v/v) Tween-20 and were then exposed to X-ray film.

### Microscopy imaging

For confocal microscopy, plant leaf samples were stained with propidium iodide (10 μg/ml) for 1.5 min and subsequently 4′,6-diamidino-2-phenylindole (DAPI; 4 μg/ml) for 20 min. Leaf was imaged using an Olympus FluoView1000 confocal microscope, with the following laser and filter combinations: 488-nm laser line with a 500–545 nm band-pass emission for YFP; the 559-nm laser line with a 575–675 nm band-pass emission for propidium iodide and 405-nm laser excitation and a 425–475 nm emission for DAPI. When more than two fluorochromes were imaged at the same time, the sequential laser scanning method was used to prevent crossing-over between different fluorochromes.

## RESULTS AND DISCUSSION

### Establishment and characterization of transgenic RNAi lines

To identify new factors required for RNAi, we established an inverted repeat (IR) construct corresponding to the 5′ region (‘*LU*’) of the firefly *LUCIFERASE* (*LUC*) gene driven by the phloem specific *AtSUC2* promoter. Processing of the *LU* dsRNA in companion cells triggers extensive RNAi of a *p35S:LUC* transgene carried by the same T-DNA, resulting in an almost complete loss-of-luminescent signal in transgenic *Arabidopsis* (referred to hereafter as *Parental Line* (*PL*); Supplementary Figure S1A–E). Phloem-specific expression of the *dsLU* was confirmed by introducing the *GUS* open reading frame downstream of the *pSUC2.* The resulting *pSUC2:GUS-dsLU-p35S:LUC* construct recapitulated the extensive *LUC* silencing phenotype observed in the *PL* and revealed a vein-restricted GUS staining pattern (Supplementary Figure S1F–I). Moreover, 35S promoter-driven expression of the *Tomato bushy stunt virus* P19 silencing suppressor led to a strong luminescence recovery in the *PL*, supporting post-transcriptional silencing mediated by *LU*-derived 21-nt siRNAs, for which P19 displays high and selective affinity (Supplementary Figure S1J–L) (29).

**Figure 1.**
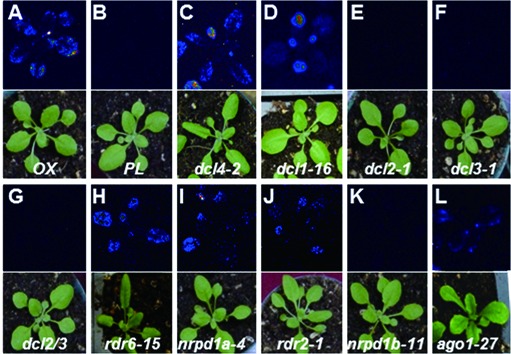
Characterization of the *PL*. (A*–*L) *LUC* silencing phenotypes in *OX* (**A**), *PL* (**B**) and different RNAi mutant [*dcl4-2* (**C**), *dcl1-16* (**D**), *dcl2-1* (**E**), *dcl3-1* (**F**), *dcl2/3* (**G**), *rdr6-15* (**H**), *nrpd1a-4* (**I**), *rdr2-1* (**J**), *nrpd1b-11* (**K**)*, ago1-27* (**L**)] backgrounds. All plants were homozygous for the silencing transgene and corresponding mutation. In *ago1-27* background, LUC activity rescue was very weak, thus the image shown here is an individual plant showing the strongest signal.

In order to determine the basic genetic requirements for *LUC* silencing, we crossed the *PL* to several mutants previously implicated in either biogenesis, movement/perception or activity of IR-derived siRNAs (16,30). We found that *LUC* silencing was relieved in the *dcl4* mutant background, but remained unaffected in the *dcl2*, *dcl3* or the double *dcl2/dcl3* mutant backgrounds, further supporting post-transcriptional silencing of *LUC* (Figure 1A–G). Moreover, luminescence was also recovered in a *dcl1* hypomorphic mutant background, agreeing with the documented role for DCL1 in optimizing the primary processing of IRs, which presumably facilitates their access and processing by other DCLs (16,31).

Extensive silencing of transgenes, as opposed to endogenes, usually entails the amplification of siRNA production through RDR6 and downstream DCL activities. These amplified secondary siRNAs can also spread from their sites of biogenesis to the neighboring cells, resulting in amplified silencing movement (32,33). As expected from the transgenic nature of the target transcript in the *PL*, extensive *LUC* silencing was fully dependent on RDR6 activity (Figure 1H). In agreement with previous findings, luminescence in the *PL* was also recovered, although to a slightly lower extent, in *nrpd1a* and *rdr2* mutant backgrounds, but not in the *nrpd1b* mutant background (Figure 1I–K) (16,17). Surprisingly, *LUC* silencing was only moderately impacted by the *ago1-27* mutation (Figure 1L, see legend), possibly because the *ago1-27* allele is still partially competent for endonucleolytic cleavage of target mRNAs (5). Alternatively, other AGOs might contribute cooperatively with AGO1 to silence the *LUC* mRNA in the *PL*. Collectively, these results suggest that the *LUC* silencing system has similar genetic requirements to those of previous IR-based systems designed to study both cell- and non-cell-autonomous RNAi in *Arabidopsis* (16,17,30,32).

### TSL contributes to *LUC* RNAi

To identify new components involved in plant RNAi, an EMS mutagenesis was conducted on the *PL* and four recessive mutants exhibiting luminescence recovery were identified and defined as *impaired luciferase silencing* mutants (*ils1-ils4*). The *ils2, ils3* and *ils4* mutations were rough-mapped to chromosomes 3, 4 and 4, respectively, and a genetic complementation assay indicated that *ils2* is allelic to *rdr6*, confirming the results obtained by reverse genetics (Figure 1H). We report here the detailed characterization of the *ils1* mutant.

**Figure 2.**
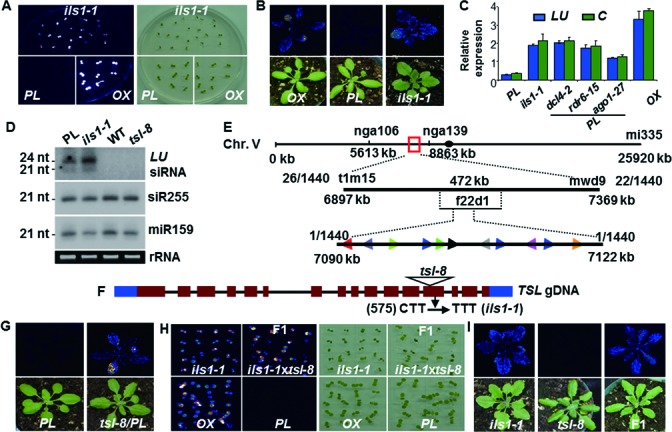
Identification and characterization of the *ils1* locus. (**A**) Luminescence image of *ils1* in 8-day-old seedlings along with *PL* and transgenic plants expressing the *p35S:LUC* transgene (overexpressor, *OX*). (**B**) Luminescence images of 3-week-old plants. (**C**) Expression of *LUC* (*LU-* and *C-*specific probes) in leaves of *PL*, *ils1*, *OX* and different RNAi mutant (*dcl4-2, rdr6-15, ago1-27*) plants in the *PL* background determined by qRT-PCR analysis. Expression levels were normalized to *ACTIN2* (*At3g18780*). Error bars indicate standard deviation for three independent experiments. (**D**) Northern-blot analysis of different small RNA accumulation in leaves from *PL*, *ils1*, WT and *tsl-8* plants. Upper panel, 21- and 24-nt *LU* siRNA; middle panel, 21-nt tasiRNA255; lower panel, 21-nt miR159; rRNA served as the loading control. (**E**) Map-based cloning of *ils1.* The *ils1* locus was mapped between the SSLP marker nga106 and nga139 on chromosome 5. Number of recombinant plants is shown for each marker. The *ils1* mutation was located on BAC f22d1, encoding several predicted genes indicated by different colored arrowheads. (**F**) Diagram of the *TSL* genomic locus. Dark red boxes indicate exons, black lines introns, blue boxes UTRs and triangle a T-DNA insertion in the *tsl-8* mutant. A missense point mutation (C to T transition) in *ils1* resulted in the amino acid substitution L575F in the 13^th^ exon of *TSL*. (**G**) Reactivation of luminescence phenotype by *tsl-8* in the *PL* background. (H–I) Luminescence and light images of *ils1*, *tsl-8* and the F1 progeny from a cross between *ils1* and *tsl-8* in seedling (**H**) and rosette stage (**I**).

Significant luminescence recovery associated with higher *LUC* mRNA levels was observed in *ils1* compared to the silenced *PL*, at both the seedling and young rosette leaf stages (Figure 2A–C). However, this increase in *LUC* transcript levels was not accompanied by decrease in the level of 21-nt or 24-nt *LUC* siRNAs (Figure 2D), indicating that *ILS1* is not required for *LUC* siRNA biogenesis or stability. Positional cloning and sequencing (Figure 2E; details in ‘Materials and Methods’ section) revealed that *ils1* carries a C→T transition mutation in the 13^th^ exon of *TSL* (At5g20930; Figure 2F). Homozygous introgression of the *tsl-8* T-DNA insertion allele (Salk_152957) into the *PL* also reactivated luminescence (Figure 2G). Moreover, all individuals of an F1 progeny from a cross between *ils1* and *tsl-8* showed the *LUC* silencing-deficient phenotype (Figure 2H–I). Therefore, *ILS1* is allelic to *TSL*.

### TSL contributes to *SULPHUR* (*SUL*) silencing

The impact of TSL on RNAi was also assessed in the *SUC:SUL* (*SS*) system in which an IR construct, driven by the *AtSUC2* promoter, directs silencing of the endogenous *SUL* mRNA (16,30). Processing of the phloem-specific *SUL* dsRNA generates 21- and 24-nt siRNAs and causes RNAi spread manifested by a chlorotic phenotype expanding 10–15 cells beyond the vasculature. Of the two siRNA species, only 21-nt siRNAs are involved in *SUL* RNAi, which, unlike in the *LUC* system, is not amplified by RDR6 owing to the endogenous nature of the silencing target (16,32). Introducing the homozygous *tsl-8* mutation in the *SS* plants led to a marked reduction in the appearance of the *SUL* silencing phenotype without affecting *SUL* siRNA accumulation (Supplementary Figure S2A–H). This observation confirmed the results obtained with the *PL* and supports the notion that TSL acts downstream of DCL4 activity in the RNAi pathway, in a process that either affects siRNA movement or siRNA activity.

Impaired movement should translate into strongly reduced siRNA levels in the *PL* where the bulk of these molecules is mainly contributed by secondary siRNA generated in an RDR6-dependent manner from the ubiquitously expressed *LUC* transgene mRNA. These move extensively throughout the entire leaf lamina, unlike the limited amount of primary siRNAs generated in the *SS* system. Therefore, the unchanged *LUC* siRNA accumulation in *tsl-8* compared to the *PL* (Figure 2D) and the fact that *SUL* siRNA levels also remain unaltered in the *tsl-8* background (Supplementary Figure S2H) favor the hypothesis that TSL is required for IR-derived siRNA activity.

### TSL acts downstream of small RNA biogenesis and is neither required for miRNA- nor endogenous siRNA-guided RISC activity

**Figure 3.**
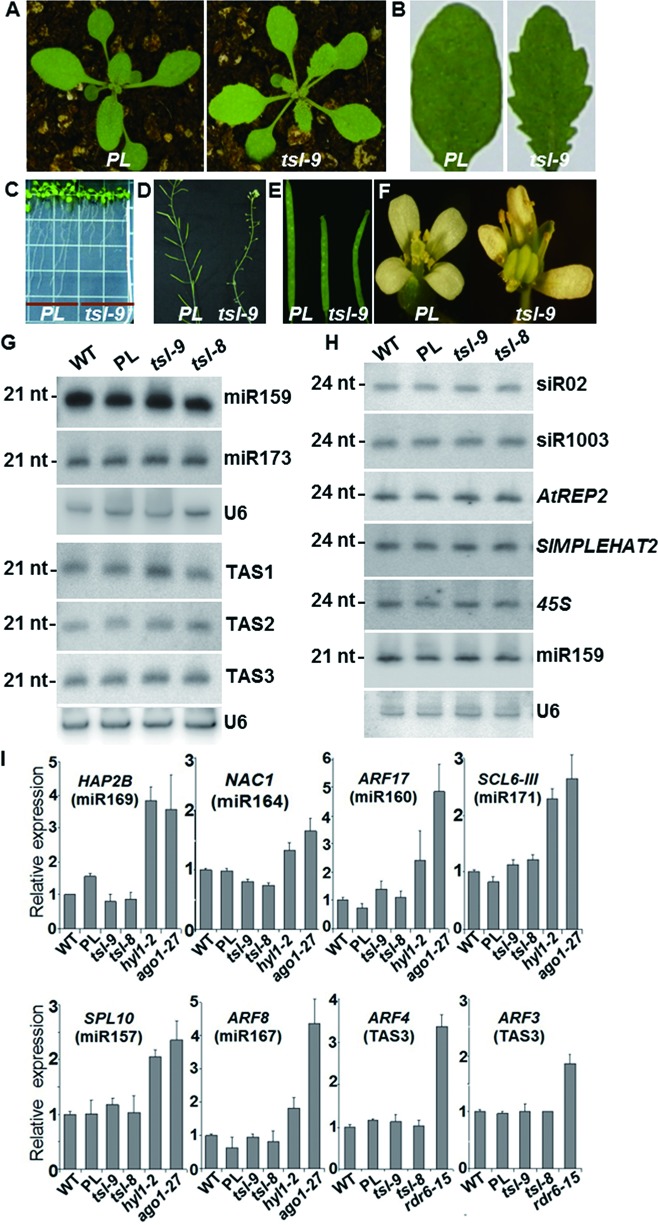
Developmental defects and analysis of endogenous small RNA accumulation or activity in *tsl* mutants. (**A**) Developmental phenotype of 18-day-old *PL* (left) and *tsl-9* mutant (right) plants. (**B**) Third rosette leaf of *PL* and *tsl-9* plants. Note the serration present at the leaf margin of the *tsl-9* mutant. (**C**) Primary root of *PL* and *tsl-9* plants. (**D**) Mature inflorescence having significantly reduced seed set in *tsl-9* compared to *PL* plants. (**E**) Siliques of *tsl-9* mutant are smaller in size than those of the *PL* plants. (**F**) Flowers of *PL* and *tsl-9*; *tsl-9* flowers lack various floral organs, such as sepals, petals, stamens and also have unfused gynoecia. (**G**) RNA blot analysis showing the accumulation of different miRNAs (miR159, miR173) and tasiRNAs (tasiR255, tasiR1511, tasiR2142) in WT, *PL*, *tsl-9* and *tsl-8* plants. U6 was used as a loading control. (**H**) RNA blot analysis showing the accumulation of different hc-siRNAs (*siR02, siR1003, AtREP2, SIMPLEHAT2, 45S*) in WT, *PL*, *tsl-9* and *tsl-8* plants. miR159 and U6 were used as loading controls. (**I**) qRT-PCR analyses to detect mRNA accumulation for different miRNA and tasiRNA target genes in WT, *PL*, *tsl-9*, *tsl-8, hyl1-2, ago1-27* and *rdr6-15* plants. Total RNA was extracted from rosette leaves for cDNA synthesis. The small RNAs that target each of these endogenous mRNAs are indicated in parenthesis. Quantification levels were normalized to *ACTIN2*, with the value from WT plants arbitrarily set to 1.0. Error bars represent standard deviation from two independent experiments in which triplicate PCRs were performed.

*TSL* was previously implicated in leaf and floral development (20). Accordingly, the *ils1* mutant (referred to hereafter as *tsl-9*) exhibited pleiotropic developmental phenotypes, such as serrated rosette leaf margins, short primary root length, reduced fertility (or complete sterility in *tsl-8*) associated with shorter siliques, as well as split and deformed gynoecia (Figure 3A–F). Given that such developmental defects are reminiscent of those exhibited by miRNA-deficient *Arabidopsis* mutants, we assessed the accumulation of various endogenous small RNAs in the *tsl* mutant background. Northern analysis showed that the levels of DCL1-dependent miRNAs in *tsl-8* and *tsl-9* mutants were comparable to those of wild-type (WT) plants, as was the accumulation of several DCL1-/DCL4-dependent tasiRNAs (Figure 3G; Supplementary Figure S3A and B). In addition, representative passenger strand of miRNA (miRNAs*) such as miR390*, miR396* and miR173* showed similar level between WT and *tsl* mutants (Supplementary Figure S3C). In contrast to guide strand miR173 (Figure 3G), RNA blot signals of miR173* were barely detectable in both the WT and *tsl* mutants, suggesting a degradation of passenger strand miRNAs*. These data indicated that unwinding of the duplex miRNA/miRNA* and their loading onto the AGO1-RISC were unaffected in *tsl* mutants. Moreover, accumulation of known miRNA and tasiRNA targets remained unchanged in these *tsl* mutants, suggesting that neither pathway requires TSL function (Figure 3I; Supplementary Figure S4).

Several transcriptional gene silencing (TGS) factors were previously implicated in cell-to-cell movement or perception of RNAi signals in recipient cells, through still elusive mechanisms (16–18). These factors include RDR2, NRPD1a and CLSY1, which are all required for production, by DCL3, of 24-nt heterochromatic (hc)-siRNA and, ultimately, for DNA methylation at the hc-siRNA producing loci (6). TSL has also been implicated in maintenance of TGS by promoting heterochromatin formation at specific loci in a DNA methylation-independent manner (34), although a role for TSL in hc-siRNA accumulation was not investigated. Therefore, we decided to monitor the levels of a set of 24-nt siRNAs produced either from polIV-dependent (*siRNA02*) or polIV/polV-dependent loci (*siRNA1003*, *AtREP2*, *SIMPLEHAT2* and *45S* rDNA). Northern analysis revealed that none of these hc-siRNAs was affected in the *tsl-8* or *tsl-9* mutant backgrounds (Figure 3H), indicating that TSL is not required for hc-siRNA biogenesis.

### TSL is required for optimal antiviral RISC function

**Figure 4.**
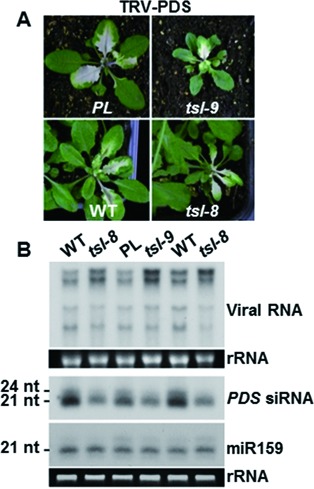
Effects of TSL on viral RNA accumulation. (**A**) Phenotypes of *TRV-PDS*-infected *PL*, WT, *tsl-9* and *tsl-8* plants. White areas result from photobleaching due to RNAi of the *PDS* mRNA triggered by *TRV-PDS* derived siRNAs. Photographs were taken 2 weeks post-inoculation. (**B**) Northern blot analysis of *TRV-PDS* viral RNAs (upper panel), virus-derived *PDS* siRNA and endogenous miR159 accumulation (lower panel) in *TRV-PDS* infected plants. rRNA was used as a loading control.

The observations made with the *PL* and the *SS* transgenic systems (Figure 2A–C; Supplementary Figure S2A–H) and the finding that neither miRNA nor tasiRNA targets are affected in *tsl* mutants suggested that TSL might be specifically required for exogenous siRNA-guided RISC function. To test if other exogenous siRNA activities were affected, viral infections were conducted on both *tsl* and control plants. For this purpose, we used a recombinant clone of *Tobacco rattle virus* (*TRV*) modified to trigger virus-induced gene silencing (VIGS) of the endogenous *PHYTOENE DESATURASE* (*PDS*) mRNA. Interestingly, although VIGS was still functional in *tsl* mutants (Figure 4A), northern analysis of RNA extracted from these infected plants showed that the *TRV* genomic RNA accumulated two-to-three fold more in the *tsl* mutant background compared to WT plants (Figure 4B), suggesting that TSL is required for optimal antiviral RISC activity. Intriguingly, this increase in viral RNA accumulation was also accompanied by a reduction in *TRV*-derived siRNA accumulation (Figure 4B). These effects could have resulted from impaired activity of RDR1, RDR2 and/or RDR6 in *tsl* mutants, all of which are required for vsiRNA production and antiviral silencing against *TRV* (35). However, this is unlikely as RDR6-dependent tasiRNA levels, *LUC* siRNA production and RDR2-dependent hc-siRNAs accumulation remained unaffected in *tsl* mutants, suggesting unaltered functionality of both polymerases (Figure 2D; Figure 3G and H).

We also considered the possibility that mutations in *tsl* might have compromised the activity/accumulation of AGO2, which was recently shown to play a role in antiviral defence cooperatively with AGO1 (36–38). Although we cannot rule out an effect on AGO2 activity *per se*, both AGO1 and AGO2 levels in the *tsl-8* mutant were comparable to those of WT plants (Supplementary Figure S5A). Moreover, *tsl* impaired *SUL* silencing, which is independent of AGO2 activity (Supplementary Figure S2A–F; PD and OV unpublished observation). Lastly, the enhanced virus levels previously reported in the *ago2* mutant background coincided with an increase, rather than a decrease, of vsiRNA levels (36). Therefore, taken together, these results suggest that the increased viral titer observed in *tsl* mutants results from suboptimal activity of the antiviral AGO1-containing RISC. In line with this idea, hypomorphic *ago1* mutations have been previously shown to cause a similar increase in viral RNA accumulation and decrease in vsiRNA levels (36,39).

### The TSL kinase activity is required for *LUC* RNAi

**Figure 5.**
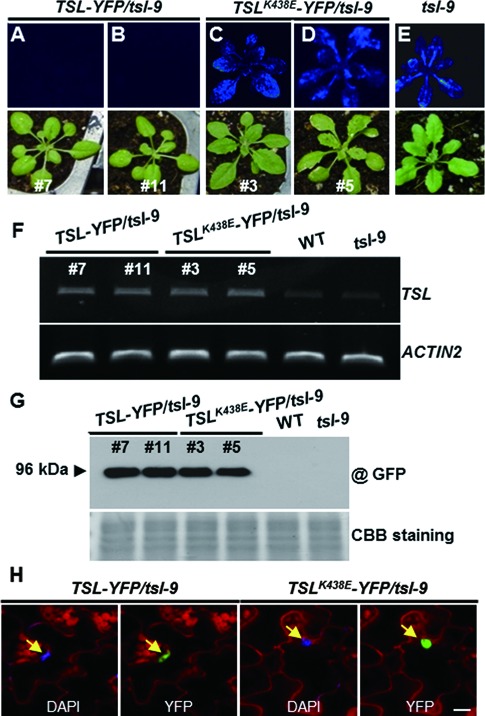
TSL kinase activity is required for *LUC* silencing. (**A**–**E**) *LUC* silencing phenotype in *35S* promoter driven transgenic *TSL-YFP/tsl-9* (A–B), transgenic *TSL^K438E^-YFP*/*tsl-9* (C-D) and *tsl-9* (E) plants. (**F**) Expression of *TSL* in plants shown in (A–E) analyzed by RT-PCR (upper panel). *ACTIN2* was used as a loading control (lower panel). (**G**) Western blot analysis using total protein extracted from leaves of the indicated plant lines using an anti-GFP monoclonal antibody (upper panel). Coomassie Brilliant Blue (CBB) staining of the membrane as a loading control (lower panel). (**H**) Nuclear localization of TSL and TSL^K438E^. Nuclei are indicated by arrows. Size bar = 10 μm.

A single point mutation that converts the conserved Lys-438 into Glu (K438E) abolishes the kinase activity of TSL without impairing its stability (22,40). To test if this activity is required for *LUC* silencing, we transformed the *tsl-9* mutant with a transgene constitutively expressing either the WT or the mutated (K438E) version of *TSL* (*TSL^K438E^*); two independent lines for each transgene were selected for further analysis. The WT *TSL* transgene successfully rescued the *LUC* silencing-deficient phenotype of *tsl-9* (Figure 5A–E), whereas transgenic lines expressing the *TSL^K438E^* allele at comparable levels still exhibited luminescence (Figure 5F and G). We confirmed that the inability of *TSL^K438E^* to restore *LUC* silencing was not due to an impaired nuclear localization of the protein (Figure 5H) (22). Collectively, these results indicate that extensive silencing of the *LUC* mRNA in the *PL* requires an intact TSL kinase activity.

Our findings strongly suggest that catalytic, as opposed to structural features of TSL are required for its RNAi-related functions (Figure 5). Two studies have identified several phosphorylation sites on human AGO proteins that are implicated in protein localization or regulation of small RNA binding (41,42). Therefore, one attractive hypothesis is that TSL might modulate the activity of specific RISCs through phosphorylation of particular AGO1 residue(s) that may alter its subcellular localization and/or the recruitment of specific AGO1-interacting cofactors. The nuclear localization of TSL is not at odds with this hypothesis, given than AGO1 localizes in both the cytoplasm and the nucleus of WT *Arabidopsis* cells (43,44). Alternatively, AGO1-interacting cofactor(s) may require TSL-mediated phosphorylation in order to interact with the exogenous siRNA-loaded RISC and promote its activity. For instance, in animal systems, RISC components such as the VASA INTRONIC GENE (VIG) or the FRAGILE X MENTAL RETARDATION PROTEIN (FRMP) were reported to be phosphorylated (45,46).

TSL was previously shown to interact with ANTI-SILENCING FUNCTION 1B (ASF1B) and the SANT/myb-domain protein, TSL-KINASE INTERACTING PROTEIN 1 (TKI1) and phosphorylate them (40). *LUC* silencing remained unaffected in the homozygous double mutant, *asf1ab* (the two *Arabidopsis* homologues of the yeast and animal *ASF1* genes) (47) or the *tki1–1* mutant background (Supplementary Figure S5B–E), suggesting that neither of these TSL substrates plays a role in the silencing deficient phenotype observed in *tsl.* In *Caenorhabditis elegans* and yeast, TOUSLED-LIKE KINASE-1 (TLK-1) is a substrate activator of the AURORA B KINASE (48), but in a manner independent of the TLK-1 kinase activity (48,49), making it unlikely that a putative plant Aurora B homolog contributes to the RNAi defects of *tsl* mutants. A final possibility is that TSL signaling indirectly regulates the expression of one or several AGO1-interacting proteins as was shown in the control of human AGO2 expression through MAP kinase signaling (50)

## CONCLUSION

It is likely that most generic or core components of the various plant small RNA pathways have been identified over the past 15 years of investigations. Consequently, new and more elaborated genetic screens are now required to isolate regulators of RNAi. Here, we have identified a plant factor required for RNAi, the protein kinase TSL that seems to act, directly or indirectly, on specific AGO1-containing RISC complexes. Indeed, although TSL seems to be required for exogenous siRNA activity (IR- and vsiRNA; Figures 2 and 4), it is apparently not necessary for endogenous small RNA-mediated regulation (miRNA, tasiRNA and hc-siRNA; Figure 3; Supplementary Figure S4). This specific effect is in line with recent findings that AGO1 might be partitioned into distinct pools, each binding preferentially to one specific small RNA class (51) and suggests that TSL may be defined, at least partly, as another layer of specificity between endo- and exo-siRNA loaded RISC. The observations that VIGS of the *PDS* mRNA was reduced but still functional in *tsl* mutants (Figure 4), and that faint *SUL* silencing remained visible in leaves of *SS* x *tsl-8* plants (Supplementary Figure S2) suggest that TSL mediates a phosphorylation event that facilitates, but is not mandatory for exogenous siRNA-loaded RISC activity. Most likely, this facilitating effect would have gone unnoticed in transgenic systems using strong inducers of RNAi, as previously reported in studies of HUA ENHANCER 1 (16,19,21).

It is becoming increasingly apparent that post-translational modifications that are embedded into signaling cascades to reprogram gene expression regulate RNAi components. In animals, prolyl-4-hydroxylation, symmetric arginine dimethylation and polyADP-ribosylation have been shown to alter stability or activity of AGO or PIWI proteins, under both normal or stress conditions (52). In plants, ubiquitination of AGO1 leads to its selective degradation through autophagy (53), whereas the CPL1 phosphatase might dephosphorylate HYPONASTIC LEAVES 1 (HYL1), the dsRNA binding partner of DCL1, to promote accurate and optimal processing of miRNA duplexes (54). Although it is still unknown whether TSL affects directly or indirectly AGO1 activity, our findings suggest that this kinase is an integral part of the signaling cascade that regulates exogenous small RNA-loaded AGO1 in *Arabidopsis*. The challenge ahead is now to identify the substrate(s) phosphorylated by TSL and to elucidate the precise regulatory function(s) of such protein(s) in the RNAi pathway.

## SUPPLEMENTARY DATA

Supplementary Data are available at NAR Online.

SUPPLEMENTARY DATA
