# Supplementary material for: The protein kinase TOUSLED facilitates RNAi in Arabidopsis
Source: Nucleic Acids Res. 2014 Jun 11;42(12):7971–80. doi: 10.1093/nar/gku422 (PMC4081062; doi:10.1093/nar/gku422)
Supplement: SUPPLEMENTARY DATA [file supp_42_12_7971__index.html]

The protein kinase TOUSLED facilitates RNAi in Arabidopsis — SUPPLEMENTARY DATA 

# The protein kinase TOUSLED facilitates RNAi in *Arabidopsis*

## SUPPLEMENTARY DATA

**Files in this Data Supplement:**

- Supplementary Figures and Table
