# Supplementary material for: The protein kinase TOUSLED facilitates RNAi in Arabidopsis
Source: Nucleic Acids Res. 2014 Jun 11;42(12):7971–80. doi: 10.1093/nar/gku422 (PMC4081062; doi:10.1093/nar/gku422)
Supplement: SUPPLEMENTARY DATA [file supp_gku422_nar-00485-a-2014-File007.pdf]

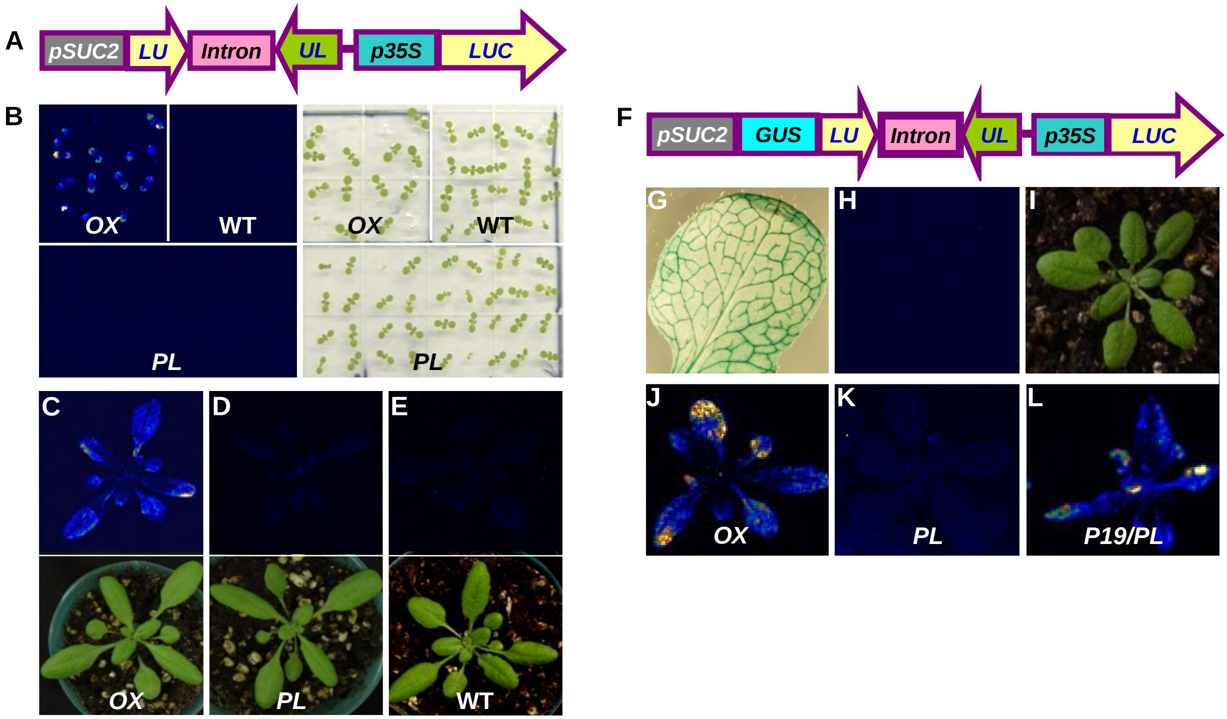

Supplementary Figure S1. A phloem-based RNAi system to identify mutants defective in spread of RNAi signal. (**A**) A T-DNA (*pSUC2:dsLU-p35S:LUC*) construct used for generating silencing parental lines (*PL*). The construct was inserted into a binary vector, pC2300 containing a kanamycin resistance gene. The PDK intron was subcloned from pHannibal. (**B**) Extensive spread of RNAi induced by a *dsLU* inverted-repeat. Luminescence images of transgenic lines expressing the construct shown in (A) (*PL*), as well as *p35S:LUC* (*OX*) lines and non-transgenic WT plants. Luminescence imaging was performed on 10-day-old seedlings**.** (**C-E**) Luminescence images and phenotypes of 3-week-old transgenic (*OX*, *PL*) and WT plants. (**F**) A T-DNA of *pSUC2:GUS-dsLU-p35S:LUC* in which the *GUS* reporter was fused downstream of the *AtSUC2* promoter. (**G**) Vein-specific *GUS* expression detected in leaves harboring the construct shown in **(**F**).** (**H,I**) Luminescence image and phenotype of plants expressing the construct shown in **(**F**).** (**J-L**) Luminescence images of *OX*, *PL* and *PL* plants expressing silencing suppressor P19.

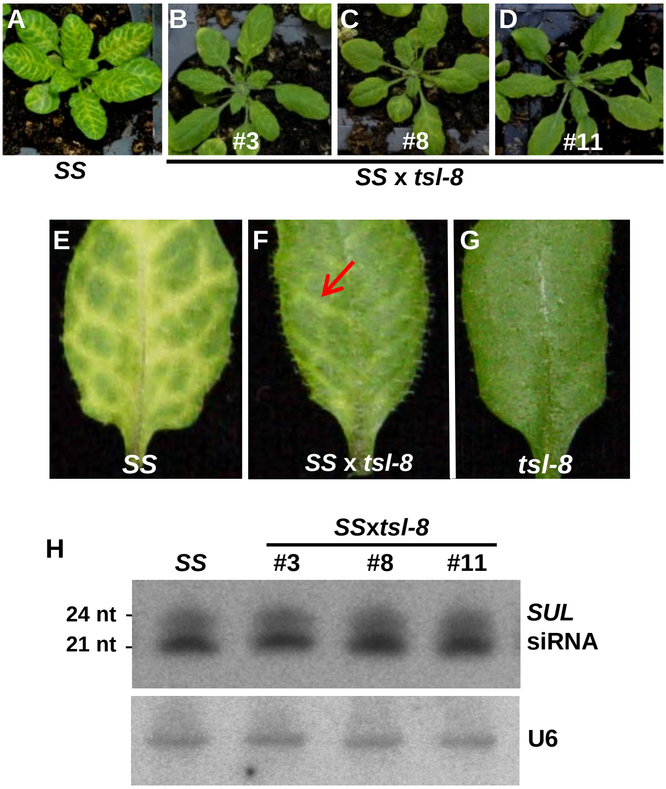

Supplementary Figure S2. Effect of *tsl-8* on RNAi in *SS* transgenic plants**.** (**A-G**) *SUL*-silencing phenotype in transgenic *SS* plant lines in *tsl-8* background. Note the weak *SUL* silencing phenotype remaining in the *tsl-8* leaves (red arrow). (**H**) RNA blot analysis of *SUL* siRNA in plants shown in A-D. U6 was used as the loading control.

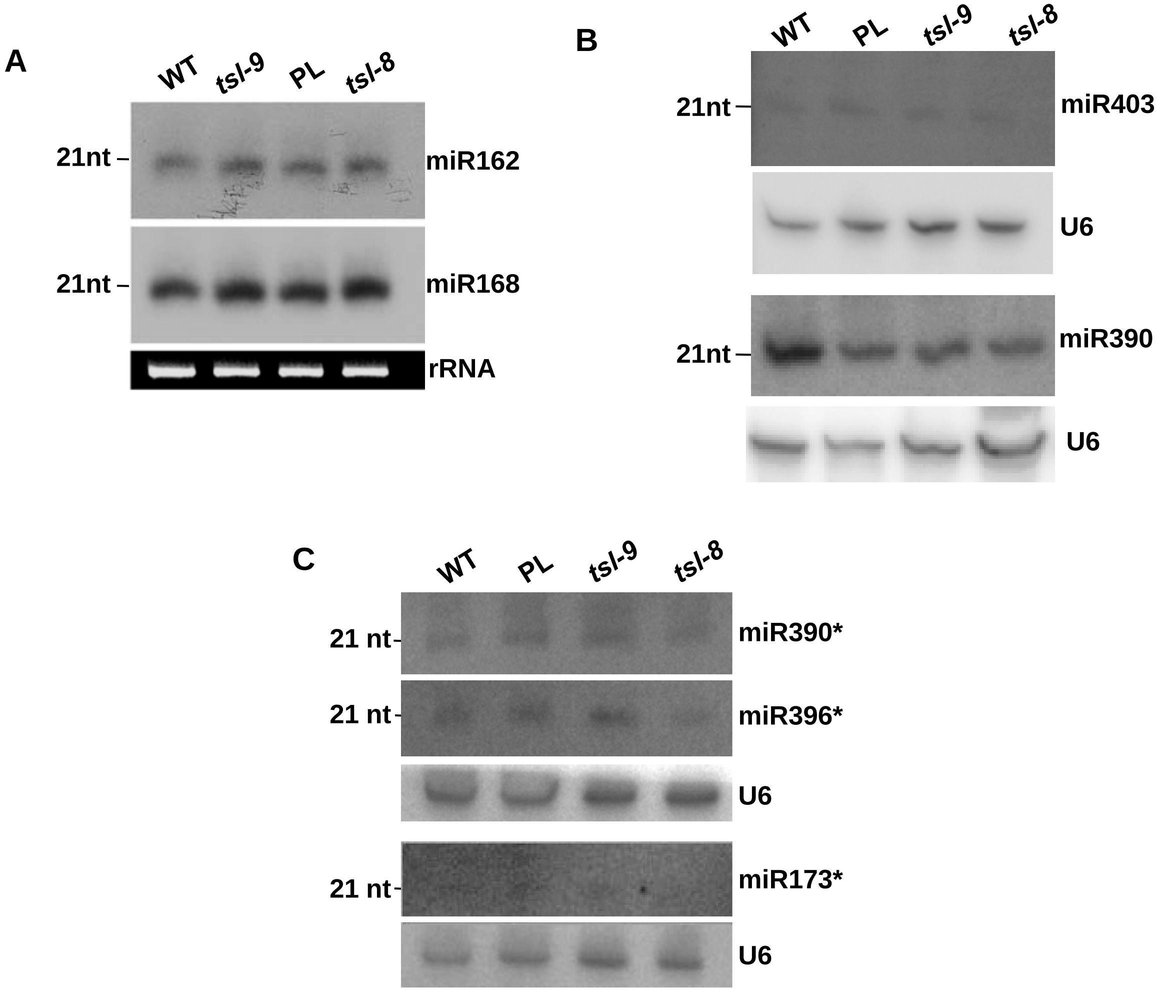

Supplementary Figure S3. TSL is not required for miRNA accumulation. (**A-C**) RNA blot analysis showing the accumulation of different miRNAs (miR162, miR168, miR403, miR390) and miRNA* (miR390*, miR396* and miR173*) in WT, *PL*, *tsl-9* and *tsl-8* plants. U6 and ribosomal RNA (rRNA) was used as the loading control.

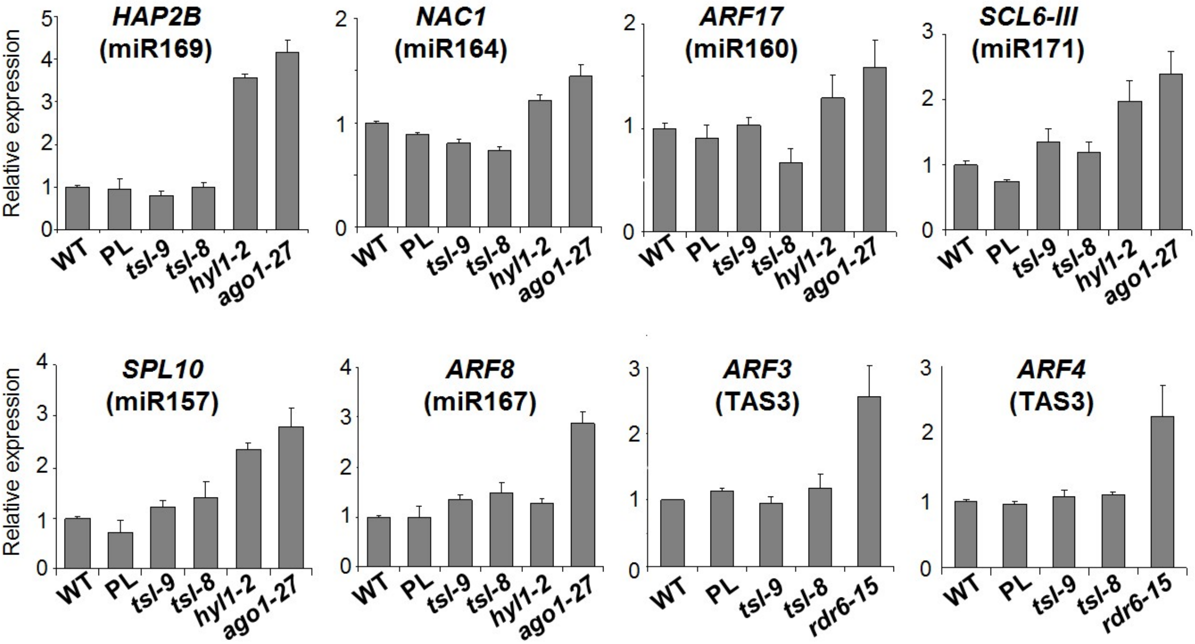

Supplementary Figure S4. The *tsl* mutation does not affect small RNA target gene expression. qRT-PCR analyses were used to assay mRNA accumulation for different miRNA and tasiRNA target genes in WT, *PL*, *tsl-9*, *tsl-8, hyl1-2*, *ago1-27* and *rdr6-15* plants. Total RNA was extracted from flowers for cDNA synthesis. The small RNAs that target each of these endogenous mRNAs are indicated in parenthesis. Quantification levels were normalized to *ACTIN2*, with the value from WT plants arbitrarily set to 1.0. Error bars represent standard deviation from two independent experiments in which triplicate PCRs were performed.

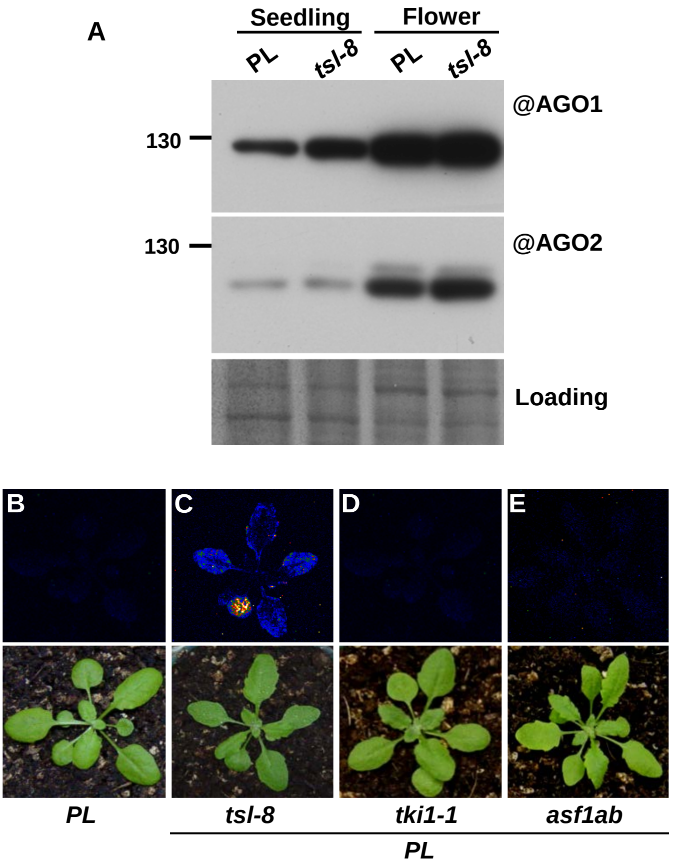

Supplementary Figure S5. Steady-state levels of AGO1 and AGO2 in *tsl-8* and identified TSL interacting proteins showing no involvement in *LUC* RNAi. (**A**) Western blot analysis using total protein extracted from seedling and floral tissue of the indicated plant lines using AGO1 and AGO2 specific polyclonal antibody. Equal loading was verified by CBB staining of the membrane after western blotting. (**B-E**) Luminescence and light images of *PL* (B), *tsl-8* (C), *tki1-1* (D) *asf1ab* double (E) mutant backgrounds.

Table S1. List of primers used in this study

Primer name	Sequences (5'-3')	Purpose
LBa1	TGGTTCACGTAGTGGGCCATCG	genotyping
tsl-8-LP	GATATGGATCCTGTGCCATTG	genotyping
tsl-8-RP	AAGCAGGCATATGATTTGGTG	genotyping
tki1-LP	AAGGGTTCCATTTGCGTAAAG	genotyping
tki1-RP	GAGAAAGTGGAGACTTTCCGG	genotyping
asf1a-2 LP	TCTCTCCCGAGTTTGGATGAA	genotyping
asf1a-2 RP	AATAGTTCTCAATTCTCATCCAC	genotyping
asf1b-1 LP	GTGAATCCATTCCAGTTCGAG	genotyping
asf1b-1 RP	CAAAACCTTGGTAGGAGGCTC	genotyping
TSL-d1	CACCATGTCGGATGACATGGT	Cloning
TSL-r1	CTTCTTAGAGTAGGCAAGATATGGATC	Cloning
TSL K438E-d1	GTGCGAGCTTCATGGTTTAAATGC	Cloning
TSL K438E-r1	AAGCTCGCACGCAACATATCTATGGTCCAC	Cloning
Luc-d1	ATGGAAGACGCCAAAAACAT	Cloning
Luc-r1	TTGGAAACAAACACTACGGTAG	Cloning, LU probe
Luc-d2	CCAGGGATTTCAGTCGATGTA	Cloning
Luc-r2	TTACAATTTGGACTTTCCGC	Cloning, C probe
LUC-d3	AATTCTTTATGCCGGTGTTG	LU probe
LUC-C-d	TTGTGGACGAAGTACCGAAA	C probe
siRNA02	GTTGACCAGTCCGCCAGCCGAT	Northern
45SsiRNA	GTCTGT TGGTGCCAAGAGGGAAAAGGGCTAAT	Northern
SimpleHat2	TGGGTTACCCATTTTGACACCCCTA	Northern
siRNA1003	ATGCCAAGTTTGGCCTCACGGTCT	Northern
siRNA255	TACGCTATGTTGGACTTAGAA	Northern
siRNA1511	AAGTATCATCATTCGCTTGGA	Northern
AtREP2	GCGGGACGGGTTTGGCAGGACGTTACTTAAT	Northern
miR390	GGCGCTATCCCTCCTGAGCTT	Northern
miR162	CTGGATGCAGAGGTTTATCGA	Northern
miR168	TTCCCGACCTGCACCAAGCGA	Northern
miR403	CGAGTTTGTGCGTGAATCTAA	Northern
miR390	GGCGCTATCCCTCCTGAGCTT	Northern
miR173	GTGATTTCTCTCTGCAAGCGAA	Northern
miR159	TAGAGCTCCCTTCAATCCAAA	Northern
ACT2-d1	TCAATCATGAAGTGTGATGTGG	RT-PCR
ACT2-r1	TTAGAAACATTTTCTGTGAACGAT	RT-PCR
